# Assessment and Incidence Determination of Myalgic Encephalomyelitis/Chronic Fatigue Syndrome Following a SARS-CoV-2 Infection in a Prospective Cohort of Hospital Employees

**DOI:** 10.3390/medicina62030480

**Published:** 2026-03-03

**Authors:** Matthias Tack, Rosalie Gruber, Leia Betting, Swetlana Herbrandt, Shuling Wu, Barbara Schlößer, Peter Häussermann, Marc Maegele, Gerlinde Schlang, Frauke Mattner

**Affiliations:** 1 Institute of Hygiene, Cologne Merheim Medical Center, University of Witten/Herdecke, Ostmerheimerstraße 200, 51109 Cologne, Germanymattnerf@kliniken-koeln.de (F.M.); 2 Statistical Consulting and Analysis, Center for Higher Education, TU Dortmund University, Vogelpothsweg 78, 44227 Dortmund, Germany; 3 Institute of Transfusion Medicine, Cologne Merheim Medical Center, Ostmerheimerstraße 200, 51109 Cologne, Germany; 4 Department of Geriatric Psychiatry and Psychotherapy, LVR Hospital Cologne, Wilhelm-Griesinger-Straße 23, 51109 Cologne, Germany; peter.haeussermann@lvr.de; 5 Department of Traumatology and Orthopedic Surgery, Cologne Merheim Medical Center, Institute for Research in Operative Medicine (IFOM), University of Witten/Herdecke, Ostmerheimerstraße 200, 51109 Cologne, Germany; marc.maegele@uni-wh.de; 6 Department of Occupational Medicine, Cologne Merheim Medical Center, Ostmerheimerstraße 200, 51109 Cologne, Germany

**Keywords:** SARS-CoV-2, Myalgic encephalomyelitis/chronic fatigue syndrome (ME/CFS), Post-COVID-19 syndrome, Healthcare worker, Nosocomial, Fatigue

## Abstract

*Background and Objectives*: Post-COVID-19 syndrome (PCS), characterized by persistent fatigue, can develop after a SARS-CoV-2 infection. Myalgic encephalomyelitis/chronic fatigue syndrome (ME/CFS) is a chronic, post-infectious condition marked by severe fatigue and post-exertional malaise. This study aimed to determine the incidence and characteristics of PCS and ME/CFS in a cohort of hospital employees (HEs) with SARS-CoV-2 infections. *Materials and Methods*: All HEs who tested SARS-CoV-2-positive between March 2020 and May 2021 who later reported persistent fatigue were invited for an assessment from July to December 2022. Canadian Consensus Criteria were used for the diagnosis of ME/CFS. Assessments included the Montreal Cognitive Assessment (MoCA), and determination of coagulation factors, Epstein–Barr virus (EBV) antibodies and autoantibodies (AABs) against G-protein-coupled receptors (GPCRs). *Results*: Of the 221 HEs, 11.8% (95% confidence interval (CI95%) 7.8–16.8, 26/221) still reported persistent fatigue and 3.2% (CI95% 1.3–6.4, 7/221) were diagnosed with ME/CFS. In total, 19 HEs (median age 51.0 years, 89.4% female, 63.1% possible or confirmed nosocomial infection) participated in our assessment. In 42.1% (8/19) MoCA results were below normal. Laboratory values showed increased GPCR AABs in 66.6% (12/18), possible EBV reactivation in 86.7% (13/15) and coagulation parameters suggesting inflammatory processes in 38.9% (7/18). *Conclusions*: Our study was able to determine lower-bound incidences of PCS with fatigue and ME/CFS and demonstrated a diagnostic pathway for HEs following SARS-CoV-2 infections. Possible EBV reactivation, increased GPCR AABs and potential coagulation cascade activation may play a pathogenic role.

## 1. Introduction

Infections with severe acute respiratory syndrome coronavirus (SARS-CoV-2) led to more than 777 million people being diagnosed with coronavirus disease 2019 (COVID-19) and more than 7 million deaths in a worldwide pandemic [1]. After a mild acute infection, 10–30% of individuals develop long-lasting symptomatology [2,3]. Conservative estimations demonstrate that 65 million people are affected by these consequences worldwide, but the actual number is believed to be higher [4]. The term long COVID is commonly used to describe persistent or new symptoms following acute SARS-CoV-2 infection lasting for more than four weeks [5]. The World Health Organization (WHO) has defined post-COVID-19 condition or post-COVID-19 syndrome (PCS) as a set of symptoms occurring three months after a probable or confirmed SARS-CoV-2 infection with a duration of at least two months, not explained by other reasons [6]. Symptom clusters include persistent fatigue, neurocognitive impairment, chest pain and shortness of breath [7]. PCS is a multisystemic condition with several pathophysiological mechanisms at play that are not yet well understood [4].

Myalgic encephalomyelitis/chronic fatigue syndrome (ME/CFS) is a complex and debilitating disease that limits daily activities and quality of life [8,9]. The worldwide prevalence is estimated to be 0.3–0.9% [10] and in Europe around three million people, mainly females, were affected prior to the COVID-19 pandemic [11]. The most prominent symptom is post-exertional malaise (PEM), characterized by disproportionate muscular and cognitive fatigue following cognitive, emotional, or even mild physical activity, with symptoms lasting from days to weeks [8,12]. Patients report preceding infections or infection-like symptomatology, for example, with Epstein–Barr virus (EBV) [9,13]. Dysregulated involvement of autonomic and central nervous, immune and cardiovascular systems, viral replication, and dysfunction of cellular energy metabolism and the endothelium are probable causes [8,11,13]. Elevated autoantibodies (AABs) against G-protein-coupled receptors (GPCRs) may serve as biomarkers for ME/CFS [14]. The exact mechanisms remain unclear and are subject to research globally. Due to the lack of a cure, individual treatment primarily involves “pacing”, a strategy that aims to prevent overexertion, alongside symptom management to alleviate disease manifestations [9,11]. A subset of PCS patients fulfills diagnostic criteria for ME/CFS [15,16,17].

In a closed cohort of hospital employees (HEs), Gruber et al. demonstrated that 47.1% (104/221) reported more than one persisting symptom after 90 days following a mild SARS-CoV-2 infection [18].

The aim of this study is to estimate the incidence of ME/CFS in the above-mentioned [18] cohort of HEs with persistent fatigue at least one year after a SARS-CoV-2 infection. Furthermore, the studied cohort underwent a systematic assessment adapted for HEs with possible ME/CFS, including characterization by routine laboratory values, functional tests and screening for pathogenic mechanisms.

## 2. Materials and Methods

### 2.1. Study Design

This prospective observational cohort study followed a previous study by Gruber et al. [18] and was conducted and reported according to the STROBE (Strengthening the Reporting of Observational Studies in Epidemiology) guidelines [19]. The study included HEs from three medical centers, belonging to a single hospital in Cologne (Germany), and is part of the HALE (Health Care Workers Affected by Long COVID and Exhaustion/Fatigue) study 2022.

### 2.2. Settings and Subjects

During the pandemic, the hospital’s institute of hygiene followed up on SARS-CoV-2 infections of all patients and HEs according to the German public health authorities’ instructions. Between May and July 2022, all HEs who reported persistent fatigue in the previous survey were contacted again. In cases with persisting fatigue at the time of reassessment, they were asked to participate in the study. The inclusion criteria were: (1) informed consent, (2) having tested positive between March 2020 and May 2021 as HEs and (3) persistent fatigue following the primary SARS-CoV-2 infection.

### 2.3. Assessment and Data Collection

Part of the survey was carried out as a telephone interview, with further investigations during in-person assessments. The 24 previously queried symptoms [20,21,22] were supplemented based on the patients’ responses and the current literature [23,24]. All symptoms included in the study had a new onset after the first infection and were classified by presence and duration. Transmissions of SARS-CoV-2 infections were classified as nosocomial, possibly nosocomial and community-acquired, as previously described [18].

An outpatient assessment was carried out with detailed medical history and blood sampling. Participants were diagnosed with PCS if they met the WHO criteria as described in the Introduction [6]. The diagnosis of ME/CFS was established using the Canadian Consensus Criteria (CCC) [25], applied in the German translation provided by the Charité Fatigue Center [26]. In this version, the CCC were presented as bullet-point items with dichotomous (yes/no) responses and were administered in a standardized order by a physician or a trained member of the research team (Appendix A, Table A1). The CCC assessment was based exclusively on patient-reported information obtained during the structured evaluation. No clinical interpretation beyond the explicit CCC definitions was applied, and partially fulfilled criteria were not considered sufficient for case classification. For further medical evaluation and systematic exclusion of potential differential diagnoses of PCS and, where applicable, ME/CFS, HEs directly employed by the hospital underwent additional assessment at the Department of Occupational Medicine according to a standardized protocol. HEs who were no longer employed by the hospital or who worked for subcontractors did not receive further clinical assessment at the Department of Occupational Medicine; instead, they were instructed to obtain blood samples through their primary care physician and to undergo evaluation according to the same standardized protocol.

This comprehensive medical workup included an extended medical history, physical examination, assessment of vital signs, and blood sampling to identify alternative medical explanations for the reported symptoms. When clinically indicated, participants, including those employed by subcontractors, were referred to other hospital departments, and the results of the assessments were subsequently compiled. Blood samples collected at the Department of Occupational Medicine were subjected to a complete blood count (XN-9100, Sysmex Deutschland GmbH (Hamburg, Germany)), along with basic renal, hepatic, and metabolic function tests, as well as measurements of thyroid hormones, iron and vitamin D-25-OH (Appendix A, Table A5). All laboratory analyses were performed by SYNLAB Leverkusen GmbH (Leverkusen, Germany).

The Bell-score was used to assess the current functional status [27]. An ECG recording and an orthostatic test were performed. The orthostatic test was conducted with participants initially in the supine position for eight minutes, followed by standing upright for eight minutes, and then returning to the supine position for six minutes. Each phase was strictly timed. Blood pressure (BP) and heart rate were measured at two-minute intervals using an automated oscillometric monitor (Vital Signs Monitor, VS-900, Mindray (Huntingdon, UK)) [28]. Participants were asked to report any symptoms, such as dizziness, palpitations, or lightheadedness, throughout the test; the test could be terminated if participants experienced severe symptoms. Postural orthostatic tachycardia syndrome (POTS) was defined as a heart rate increase > 30 beats per minute (bpm) or a standing heart rate > 120 bpm. Orthostatic hypotension (OH) was defined as a systolic BP decrease > 20 mmHg or diastolic BP decrease > 10 mmHg [29,30]. For the evaluation of mild cognitive impairment, the German-language version of the Montreal Cognitive Assessment (MoCA, version 7 November 2004) was used. The maximum score is 30 points, with ≥26 points indicating normal cognitive performance. An additional point was added for participants with 12 years of education or less. The test was administered by a trained examiner who had completed formal MoCA training provided by the test publisher [31]. Our assessment further included questions on the presence of burning pain in extremities, sensory disorders, sleep disorders due to pain, increase in pain at night, and hyperesthesia, asking for a yes or no response to screen for a possible small fiber neuropathy (SFN) [32]. Given the absence of confirmatory diagnostic procedures, such as skin biopsy and histology, the SFN screening results are exploratory and do not constitute a validated diagnosis of SFN.

Polymerase chain reaction (PCR) testing of a hypopharyngeal swab was performed [33] to exclude an acute SARS-CoV-2 infection. A line immunoassay (recomLine, MIKROGE GmbH (Neuried, Germany)) was used for EBV antibody serology. The analyses were performed by SYNLAB Leverkusen GmbH (Leverkusen, Germany) as part of the previously described blood sampling and assessment conducted at the Department of Occupational Medicine. The test included detection of immunoglobulin G (IgG) antibodies against Epstein–Barr nuclear antigen-1 (EBNA1), p18 and p23 virus capsid antigen (VCA), BZLF1 intermediate early antigen (IEA), p138 early antigen-restricted (EA-R) and p54 EA-diffuse (EA-D), and immunoglobulin M (IgM) antibodies against p23 VCA, ZEBRA IEA, p138 EA-R and p54 EA-D.

A blood coagulation panel was analyzed and was completed with acute phase reactants (APRs) including fibrinogen, factor VIII and von Willebrand factor (VWF), negative APR antithrombin, and homocysteine concentration. VWF was determined as antigen and activity each for blood group (BG) 0 and combined for BGs A, B and AB, later referred to as the BG categories. The analysis (ACL TOP 750, Werfen GmbH (München, Germany)) used a calibration curve generated by diluted standard plasma to convert the measured clotting time into percentages of their relative activity, whereby plasma from a pool of >30 donors corresponded to 100%. Thrombelastography was performed (ROTEM^®^ *sigma*, Werfen GmbH (München, Germany)) with FIBTEM, EXTEM, INTEM, and APTEM cartridges [34].

Blood samples were collected and frozen at −80 °C for further determination of GPCR AABs. GPCR AABs were determined by IMD Institut für Medizinische Diagnostik Berlin-Potsdam GbR laboratory (Berlin, Germany) with specific commercial sandwich ELISA kits (CellTrend GmbH (Luckenwalde, Germany)) for quantitative determination according to the manufacturer’s instructions. Eight IgG AABs, including those against angiotensin II type 1 receptor (AT1R), ß-2 adrenergic receptors (ß2-adr-R), and muscarinic acetylcholine receptor M3 (M3R) and M4 (M4R), were determined, each expressed as within normal limits, weakly increased, and increased. A detailed assessment protocol is available in Supplementary File.

### 2.4. Data Analysis

Descriptive statistics were performed for the study population and for the frequency and duration of reported symptoms. Incidences of fatigue and ME/CFS were assessed among 221 HEs who participated in the previous systematic survey [18]. Estimates were based on a staged evaluation of a subset of participants who had previously reported persistent fatigue, first contacted by telephone and later assessed in person. Incidences are reported as absolute numbers with 95% confidence intervals (CI95%). This staged evaluation allowed systematic characterization of fatigue and ME/CFS in a subset, while acknowledging that cases without prominent fatigue may not have been captured. Analyses were conducted using complete-case data for each variable. Denominators therefore vary across analyses depending on variable-specific missingness. No observations were excluded from the analyses, and no imputation of missing data was performed. Laboratory values were analyzed using descriptive statistics, and truncated values were used as absolute numbers. No *p*-values were calculated due to the small sample size. For all analyses the free statistical software R (version 4.3.1, R Core Team, 2023 (Vienna, Austria)) [35] was used.

### 2.5. Ethical Clearance

The study was conducted in line with the principles of the Declaration of Helsinki and has received ethical approval from the ethics board of Witten/Herdecke University (number 110/2022). The study was not funded and is listed in the German Clinical Trial Register (DRKS00030178).

## 3. Results

**Figure 1:**
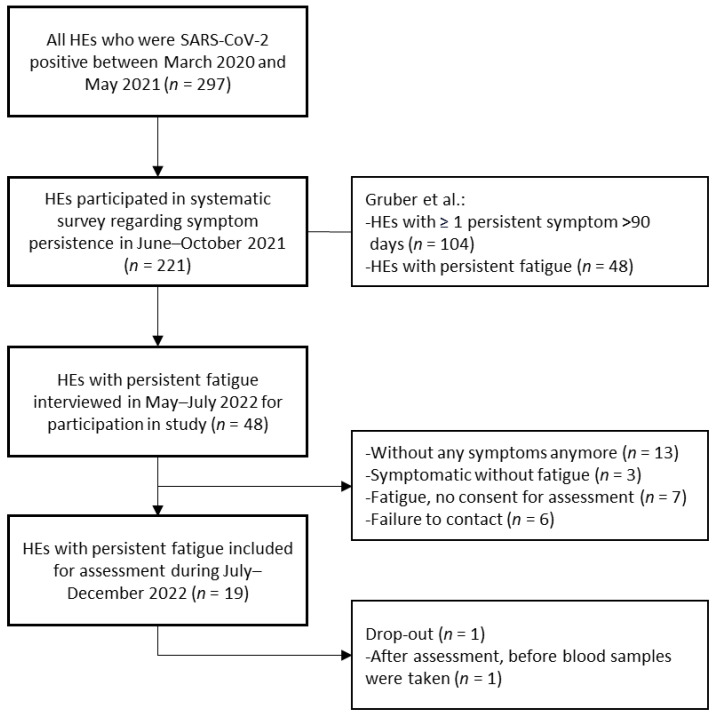
Flowchart demonstrating study participant selection. SARS-CoV-2-positive HEs that first participated in a systematic survey in 2021 by Gruber et al. [18] and later those with persistent fatigue were asked to participate in the follow-up assessment in 2022. HE, hospital employee.

After applying the inclusion criteria, 48 out of 221 HEs were contacted for participation in the telephone interview (Figure 1). During this interview, 11.8% (26/221, CI95% 7.8–16.8) reported persistent fatigue after the initial SARS-CoV-2 infection, of which 19 HEs were recruited for further assessment, later referred to as the HALE-cohort. Their mean age was 47.79 years (standard deviation (SD) 10.8) with a range of 25–61 years (Table 1). The assessment took place between July and December 2022, with a median of 629 days (interquartile range (IQR) 129, range 467 to 1008 days) after the first SARS-CoV-2 infection. Following the initial infection, 42% (8/19) reported ≥1 reinfection with SARS-CoV-2 with a total of 1–5 infections (median 1, IQR 1) until the assessment. According to WHO criteria, all HEs suffered from mild to moderate COVID-19 [36], and none of them were hospitalized during the first infection. HEs were infected by wild-type SARS-CoV-2 and the alpha variant (B.1.1.7).

### 3.1. ME/CFS and Functional Status

The clinical assessment and subsequent laboratory investigations did not reveal any alternative explanation for the reported symptoms in any participant. Accordingly, all assessed individuals (19/19) were classified as having PCS. Within the HALE-cohort, 36.8% (7/19) were classified as having PCS with a diagnosis of ME/CFS (PCS-ME/CFS) and the remaining 63.2% (12/19) were classified as having PCS without ME/CFS (PCS-non-ME/CFS, Table 1). The overall Bell-score was a median of 60 (range 40–90) out of 100, for PCS-ME/CFS it was a median of 60 (IQR 10.0, range 40–80) and for PCS-non-ME/CFS a median of 65 (IQR 12.5, range 40–90). The CCC involved 38 questions; on average, 16.37 (SD 6.85) questions were answered positively, and the highest number for a participant was 33 (Appendix A, Table A1). Extrapolation for the large cohort (*n* = 221) shows that at least 3.2% (7/221, CI95% 1.3–6.4) fulfill the CCC for a diagnosis of ME/CFS, based on a staged subset of participants: 26 participants with persistent fatigue, 19 who underwent further assessment including the CCC, and 7 who ultimately met the full criteria. Under the assumption that HEs who were not included in the detailed assessment but reported persistent fatigue (*n* = 7, Table 1), as well as those who could not be contacted (*n* = 6, Table 1), might have met ME/CFS diagnostic criteria, the estimated incidence would range from 3.2% to 9.0% (7–20/221).

### 3.2. Reported Symptoms

**Figure 2:**
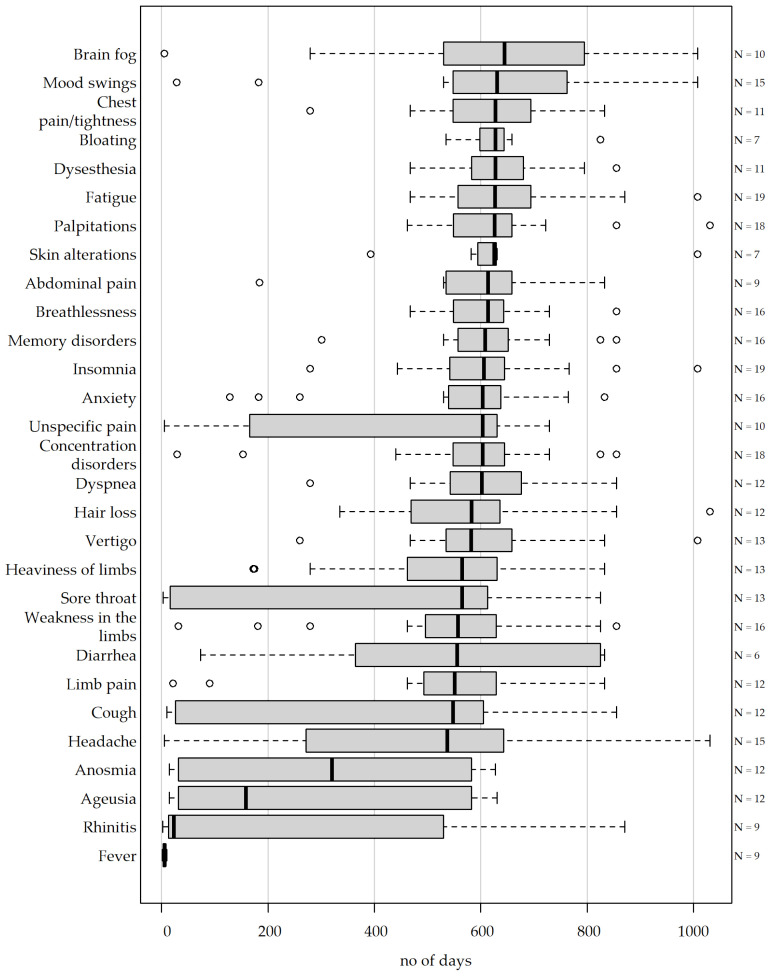
Occurrence and duration of persistent symptoms. Boxplots representing reported symptoms in hospital employees (HE, *n* = 19) after SARS-CoV-2 infection. If reported by >4 participants, the applicability and duration (in days) of all symptoms, arranged in decreasing order of median duration after initial SARS-CoV-2 infection, are visualized in these boxplots. If still ongoing, the date of the assessment was selected as the minimum duration. The number of HEs complaining of a symptom at any time is indicated on the right-hand side.

In our systematic survey, on average, 19.90 (SD 4.72) out of 33 symptoms and complications were reported to have occurred after the SARS-CoV-2 infection (Figure 2). Fatigue and insomnia were reported by every participant (19/19, Figure 2, Appendix A, Table A2). Concentration disorders and palpitations were reported in 94.7% (18/19), and memory disorders, breathlessness, weakness in the limbs and anxiety in 84.2% (16/19). These symptoms persisted for a median of at least 550 days after the initial SARS-CoV-2 infection. Fever was reported in 47.3% (9/19), with a median duration of 6 days. Nausea and subfebrile temperatures were reported in 21.1% (4/19). Persistent vomiting and a bleeding event were reported in one participant.

### 3.3. Orthostatic Test and Montreal Cognitive Assessment

In the orthostatic test, 1 HE (1/19) was diagnosed with POTS and 21.0% (4/19) with OH. The mean score of 25.63 points in the MoCA tests was below normal (SD 2.97, range 20–30 points), with 42.1% (8/19) of HEs having results below the cut-off. HEs with ME/CFS had a lower mean value of 24.90 (SD 3.18) compared to those without (mean 26.10, SD 2.87). For each category, average points were as follows: 4.21 (SD 0.85, range 2–5 out of 5 points) for visuospatial/executive, 2.89 (SD 0.32, range 2–3 out of 3 points) for naming, 5.42 (SD 0.84, range 3–6 out of 6 points) for attention, 1.84 (SD 1.12, range 0–3 out of 3 points) for language, 1.21 (SD 0.85, range 0–2 out of 3 points) for abstraction, 3.95 (SD 1.22, range 1–5 out of 5 points) for memory and 6.0 (SD 0.0, range 6-6 out of 6 points) for temporospatial orientation.

### 3.4. Putative Small Fiber Neuropathy

In the SFN screening questionnaire, 26.3% (5/19) reported burning pain in extremities, 52.6% (10/19) reported sensory disorders, 36.8% (7/19) reported sleep disorders due to pain, 26.3% (5/19) reported pain increases at night, and 47.4% (9/19) reported hyperesthesia. All five items were negated by 36.8% (7/19). On average, 1.89 (SD 1.88) items were positive. Participants with ME/CFS had higher mean results, with 3.29 (SD 1.70), compared to those without (mean 1.08, SD 1.51). A total of 71.4% (5/7) of those with ME/CFS reported all items except burning pain in extremities.

### 3.5. Coagulation Panel

**Figure 3:**
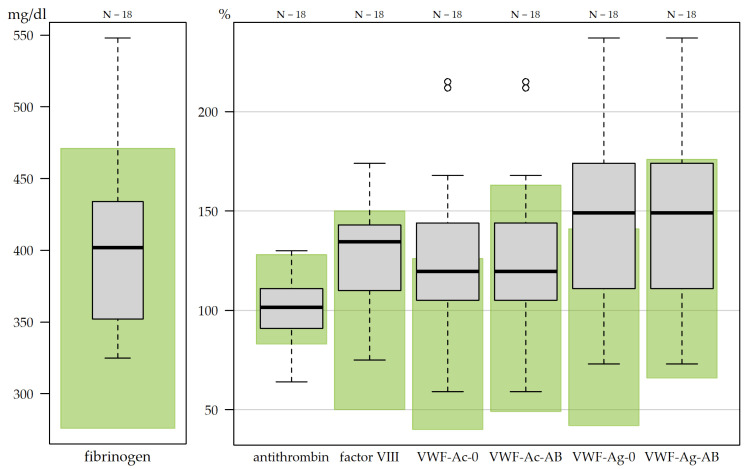
Coagulation factors in hospital employees with post-COVID-19 syndrome. Boxplots of coagulation factors, which are also considered acute phase reactants. For each coagulation factor, the respective reference range is indicated by green-shaded areas. The number of participants with available coagulation factor measurements is shown at the top of the figure. A total of 19 participants were enrolled; one participant completed the assessment but declined blood sampling, resulting in a sample size of *n* = 18 for this analysis. VWF, von Willebrand factor; VWF-Ac-0, VWF activity for blood group 0; VWF-Ac-AB, VWF activity for blood groups A, B and AB; VWF-Ag-0, VWF antigen for blood group 0; VWF-Ag-AB, VWF antigen for blood groups A, B and AB.

Electrocardiogram (ECG) recordings did not show any pathological findings (19/19). All SARS-CoV-2 PCR tests during assessment were negative (17/17). One HE completed the assessment and consented to data use but declined blood sampling. Consequently, laboratory data were available for 18 participants (Figure 1). Routine coagulation markers (Quick, International Normalized Ratio and thrombin time) showed no abnormalities, and Partial Thromboplastin Time was decreased in one HE. Determination of APR demonstrated increased fibrinogen in 16.7% (3/18), increased factor VIII in 16.7% (3/18), and decreased antithrombin in 11.1% (2/18). VWF was possibly increased in 33.3% (6/18), with increased activity or antigen in one BG category. Elevated VWF activity or antigen (≥1 increased value in each BG category) was seen in 22.2% (4/18). Possible inflammatory processes indicated by striking APR results were noted in 38.9% (7/18), as shown in Figure 3. Additionally, homocysteine concentration was 13.0 μmol/L (reference value < 10, IQR 2.3) at median and thus increased in 77.8% (7/9) of HEs (Appendix A, Table A3). One HE presented increased ferritin, also considered an APR. Thrombelastography demonstrated no major coagulopathies.

### 3.6. Autoantibodies Against G-Protein-Coupled Receptors

**Figure 4:**
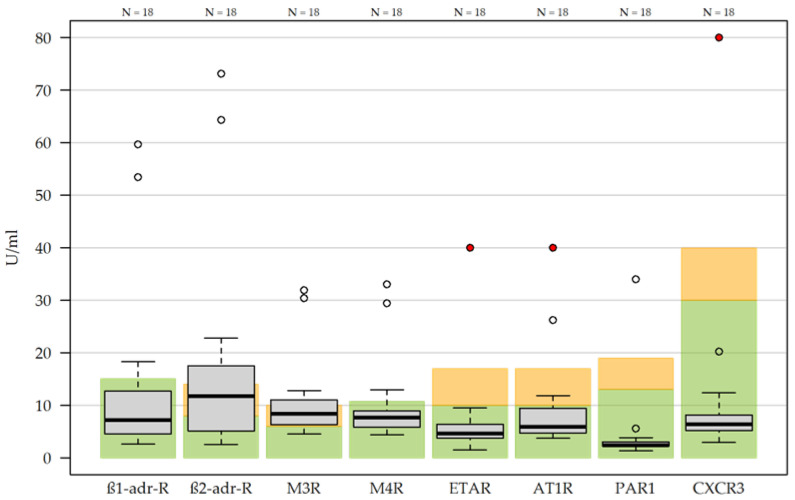
Autoantibodies (AABs) against G-protein-coupled receptors in hospital employees with post-COVID-19 syndrome, presented as boxplots. Green-shaded areas indicate the respective reference range for each AAB, representing values within the normal range. Orange-shaded areas indicate weakly increased results, and white-shaded areas indicate values above the reference range. Red dots denote values that were truncated and subsequently analyzed as absolute numbers. The number of participants with available AAB measurements is shown at the top of the figure. A total of 19 participants were enrolled; one participant completed the assessment but declined blood sampling, resulting in a sample size of *n* = 18 for this analysis. ß1-adr-R, ß-1 adrenergic receptor; ß2-adr-R, ß-2 adrenergic receptor; M3R, muscarinic acetylcholine receptor M3; M4R, muscarinic acetylcholine receptor M4; ETAR, endothelin type A receptor; AT1R, angiotensin II type 1 receptor; PAR1, protease-activated receptor 1; CXCR3, chemokine receptor CXCR3.

AAB levels (Figure 4, Appendix A, Table A3) against M3R were increased in 88.9% (16/18) and those against ß2-adr-R increased in 66.7% (12/18). AABs against M4R and AT1R were each elevated in 22.2% (4/18). The results for all eight AABs combined for each HE were considered low-grade increased in 44.4% (8/18) and increased in 22.2% (4/18) (Table 1).

### 3.7. Epstein–Barr Virus Serology

In 15 participants, EBV serology and routine laboratory parameters were assessed as part of the occupational medicine evaluation. The serological status consistent with a possible EBV reactivation was determined based on ≥1 positive IgG or IgM antibodies against EA and IEA in 86.7% (13/15). In total, 60.0% (9/15) had ≥1 positive IgG antibodies against EA-R, EA-D or BZLF1 IEA; additionally, 26.7% (4/15) had weakly positive results. In 6.7% (1/15), positive IgM against EA-R was detected and in 6.7% (1/15) weakly positive IgM against EA-D was detected. The combination of positive IgG antibodies against EBNA1 in 73.3% (11/15) and against p18 VCA in all HEs (15/15) indicates previous primary EBV infections. IgM antibodies against VCA and ZEBRA IAE were all negative (15/15), while IgG p23 VCA antibodies were all positive (15/15), which underlines that the HEs had previously undergone primary EBV infections (Appendix A, Table A4).

### 3.8. Laboratory Parameters

Increased monocyte counts were present in 40.0% (6/15). Metabolic abnormalities were shown by increased cholesterol in 53.3% (8/15), increased triglycerides in 33.3% (5/15), low HDL-cholesterol in 60.0% (9/15) and increased LDL-cholesterol in 35.7% (5/14). Thyroid hormones and iron metabolism showed minor abnormalities. Vitamin D-25-OH values were decreased in 93.3% (14/15), with mean values of 22.37 ng/mL (SD 9.55, reference range 41–100). All remaining laboratory values are shown in Appendix A, Table A5.

## 4. Discussion

In our study, at least 11% of HEs presented with ongoing fatigue and a further debilitating load of symptoms in a median of 21 months after a SARS-CoV-2 infection without prior immunization. Some of these HEs fulfill the diagnosis criteria of ME/CFS, which sums up to an incidence of at least 3% in the cohort of 221 HEs. Notably, the observed incidence is only based on those HEs who reported fatigue in the previous survey. Some of the HEs without fatigue in the previous systematic survey [18] may have also developed ME/CFS following their infection; therefore, the incidence of ME/CFS in our cohort might even be underestimated. In addition, not all participants who experienced persistent fatigue in the telephone interview could be recruited for detailed assessment, which may have led to further under-ascertainment of cases. Taken together, the observed incidence should therefore be interpreted as a conservative lower bound.

The high incidence of nosocomial and possible nosocomial transmissions might be influenced by the SARS-CoV-2 variants responsible for the initial infections, and by working in healthcare institutions during the early phase of the COVID-19 pandemic when vaccinations were not yet available. Since then, vaccinations have proven protective against the overall development of PCS [37]. While nosocomial infections have caused PCS-ME/CFS in HEs, the proportion was higher for community-acquired PCS.

Our study aims to estimate the incidence of post-COVID-19 ME/CFS in defined cohorts. Due to differing diagnostic criteria for ME/CFS, the European expert consensus recommends the 2003 CCC for research, while the 2015 Institute of Medicine (IOM) criteria are more suitable for primary care settings. The CDC-1994/Fukuda criteria, not considering PEM, are still used in some studies [11]. A study with 102 COVID-19 patients found 2.5% presenting with ME/CFS after six months using the Fukuda criteria [38]. Unger et al. found a prevalence of 2.8% of ME/CFS-like illness after 12 months in their study of 2418 SARS-CoV-2-positive participants, based on the IOM criteria [39]. Similarly, data from the RECOVER-Adult cohort demonstrated that 4.5% of 11,785 participants met the IOM criteria six months after acute SARS-CoV-2 infection [40]. These studies highlight the challenges of different diagnostic criteria within a small set of studies, yet they provide similar estimates for post-COVID-19 ME/CFS.

Our study demonstrates a diagnostic pathway specific to HEs with PCS and fatigue, starting with the listed questionnaires and applying diagnostic criteria for ME/CFS. As a further step, we aimed to exclude other differential diagnoses by medical history, laboratory values and exclusion of acute SARS-CoV-2 infections. Legler et al. have described the importance of differentiating the groups since PCS-non-ME/CFS patients presented an overall health improvement after 20 months while PCS-ME/CFS patients showed persistently higher symptom severity [16].

Pronounced neurological impairment was demonstrated by high proportions of complaints within the SFN screening questionnaire for the PCS-ME/CFS group and the overall poor MoCA test performances. Positive screening questionnaire results for SFN remain presumptive, as a definitive diagnosis requires confirmation by skin biopsy, as performed by Donadio et al. in patients following COVID-19 [41]. Besteher et al. found an increase in cortical thickness in MRI scans, which was most distinct in patients with cognitive impairment (MoCA scores < 26) and PCS [42]. Larger studies demonstrated that within six months after the SARS-CoV-2 infection, the risk of developing disturbances within nearly all organ systems increased [37]. The mentioned complexity of our participants therefore required an interdisciplinary approach. Further investigations in other departments were beyond the scope of our study.

Coagulation panels demonstrated increased APR in seven HEs, suggesting underlying inflammatory processes. Moreover, a shift in coagulation towards thrombogenic factors and elevated cardiovascular risk factors with increased homocysteine was seen, though thrombelastography did not reveal pathological patterns. Routine coagulation markers did not demonstrate any abnormalities. VWF generally demonstrated the highest proportion of elevated values, but due to the lack of blood group typing, the results cannot be analyzed in full detail. Nevertheless, increased VWF was described by Kruger et al. in PCS patients with fatigue and cognitive impairment, who were found to be suffering from microclots containing high numbers of inflammatory molecules and hyperactivated platelets [43].

Levels of AAB were at least low-grade increased in two thirds of participants, with especially high levels found for AABs against ß2-adr-R and M3R. AAB determination for 268 ME/CFS patients at the Charité Hospital in Berlin (Germany) demonstrated significantly higher AAB levels against ß2-adr-R, M3R and M4R compared to controls [44]. These results are partially similar to our study, with the exception of M4R levels. We could not detect any differences for the group of our HEs that did not fulfill the ME/CFS criteria.

Overall, the EBV serology showed 86% of participants had some degree of virus reactivation or replication. We are unable to disentangle whether reactivation of EBV might be responsible for the clinical presentation of the participants. Nonetheless, our results seem to fit with published studies and reviews in this field; however, evidence is limited [4,13]. Increased monocyte levels in the participants support the theory of reactivation.

Apart from the general metabolic risk factors observed in the blood samples, no atypical values were identified that could account for the symptomatology. The extremely low levels of vitamin D-25-OH represented an unexpected finding, particularly when compared to studies including PCS participants with and without ME/CFS, in which the median values of both groups were within the normal range [15]. However, low vitamin D-25-OH levels should be interpreted with caution, as they are influenced by seasonal variation, supplementation, and the high baseline prevalence of vitamin D deficiency in the general population. Heterogeneous findings have been reported, including significantly lower vitamin D levels in PCS patients compared to controls [45]. This finding is not disease-specific or causal.

The primary suspected mechanisms underlying PCS were seen in our cohort: Immune dysregulation was evidenced by EBV reactivation, neuroinflammation, and possible dysfunctional neurochemical signaling pathways that may underlie dysautonomia symptoms. Furthermore, the SFN screening questionnaires, together with the presence of OH and POTS in the orthostatic test, may strengthen this hypothesis. Autoimmunity aspects were shown by elevated GPCR-AABs, and excessive blood clotting was reflected in the coagulation panel results. Underlying inflammation may contribute to the mechanisms mentioned above [4]. Given the diverse pathophysiological mechanisms and subgroup overlap in PCS, multiple parameters are likely more suitable for diagnosis and understanding underlying mechanisms than a single biomarker.

### 4.1. Study Strengths and Limitations

Despite the small cohort, the main pathophysiological phenomena of PCS were detectable in a hospital setting. Lower bounds of post-COVID-19 ME/CFS were identified in a defined cohort using the most appropriate diagnostic criteria for clinical studies. Our study design reduces selection bias, as many PCS center publications lack defined cohorts [15,16]. The cohort spans a broad age range of working individuals, despite being hospital-based, and includes a homogeneous group in terms of SARS-CoV-2 infection severity, variants, and unvaccinated status, minimizing confounders. Our study demonstrates a diagnostic pathway for HEs with fatigue following SARS-CoV-2 infection.

The main limitation is the small sample size, which was limited to the number of HEs. Accuracy could have been improved by including HEs without fatigue. Despite the extended observation period, the reported incidence and symptoms may have evolved, with courses that can be stable or fluctuating. Therefore, the findings should be interpreted in the context of the time of recurrence. The lack of a control group does not allow a definite correlation between EBV serology and PCS. Subjective reports by participants were therefore not validated by controls. Due to the small sample size, it was not possible to distinguish pathophysiological phenomena specific to ME/CFS from those associated with general PCS. Furthermore, the small sample size precluded additional stratification of results by vaccination status, reinfections, or time since the primary infection and assessment, although these factors may influence symptom severity and biomarker profiles.

### 4.2. Implications for Clinical Practice and Research

A precise diagnosis is crucial for monitoring disease progression and identifying treatment options, like pacing strategies, in PCS and ME/CFS. The study underlines the impact of SARS-CoV-2 infections on PCS and post-COVID-19 ME/CFS, highlighting the need for infection prevention measures. Infection control and occupational medicine departments should be integral in managing post-infectious syndromes, given the high sickness rate in healthcare settings. Our study contributes to the global search for diagnostic markers in PCS and ME/CFS, with a particular focus on the high prevalence of EBV reactivations as a potential target for treatment approaches.

## 5. Conclusions

In summary, our study found an incidence of at least 11.8% (CI95% 7.8–16.8) for PCS with fatigue and 3.2% (CI95% 1.3–6.4) for post-COVID-19 ME/CFS in HEs 21 months after the first SARS-CoV-2 infection. We established a diagnostic pathway for severely affected HEs, where the diagnosis of ME/CFS enables more precise management. Possible EBV reactivation, increased GPCR AABs, and potential coagulation cascade activation may play a pathogenic role. This study emphasizes the need for infection prevention measures to protect HEs from nosocomial post-COVID-19 ME/CFS.

## Figures and Tables

**Table 1:** Characteristics of the participants and the main results for all and those fulfilling the CCC.

	Total *		CCC Fulfilled *	
	n	%	n	%
Total number	19	100	7	100
**Demographics**				
Females	17	89.5	6	85.7
Males	2	10.5	1	14.3
**Age**				
<40 years	5	26.3	2	28.6
≥40 years	14	73.7	5	71.4
**Professional group**				
Nursing staff	14	73.6	4	57.1
Medical staff	1	5.3	0	0.0
Others	4	21.1	3	42.9
**Transmission type**				
Community-acquired	7	36.8	4	57.1
Possibly nosocomial	5	26.3	1	14.3
Definitely nosocomial	7	36.8	2	28.6
**COVID-19 vaccinations**				
Unvaccinated at first SARS-CoV-2 infection	19	100	7	100
At time of assessment:				
1–2 vaccinations	10	52.6	4	57.1
3–4 vaccinations	8	42.1	3	42.9
NA	1	5.3	0	0.0
**SARS-CoV-2 reinfection**				
1–2 infections in total	17	89.5	7	100
≥3 infections in total	2	10.5	0	0.0
Main results *				
**Occupational medicine (n = 15) ^†^**				
Notification of OD	8	53.3	2	50.0
Confirmed OD	1	6.7	0	0.0
Treatment by ELIA	2	13.3	1	25.0
Underwent rehabilitation	2	13.3	2	50.0
**Orthostatic test (n = 19)**				
Orthostatic hypotension	4	21.1	1	14.3
POTS	1	5.3	0	0.0
**Coagulation panel (n = 18)**				
APR out of reference range	7	38.9	3	42.9
**GPCR AABs (n = 18) ^‡^**				
No increase	2	11.1	1	14.3
Selective low-grade increase	4	22.2	1	14.3
Low-grade increase	8	44.4	3	42.9
Increase	4	22.2	2	28.6
**EBV serology (n = 15)**				
≥1 positive IgG or IgM against IEA and EA of EBV	13	86.7	4	57.1

* Percentages were initially calculated based on the entire HALE-cohort and subsequently for the total number of participants fulfilling the CCC. In the results section, percentages refer to the number of participants available for each variable. Sample sizes vary due to missing data. ^†^ Participants deemed suitable for an OD diagnosis by the Department of Occupational Medicine were notified to the ELIA for confirmation of the diagnosis and to receive support in their treatment, including sending participants to a rehabilitation program. ^‡^ Combined results for eight determined GPCR AABs per participant were classified in four ascending levels: no increased AABs (all within normal limits), selectively low-grade increased AABs (=1 weakly increased), low-grade increased AABs (>1 weakly increased), increased AABs (≥2 increased). CCC, Canadian Consensus Criteria; OD, occupational disease; ELIA, employers’ liability insurance association; POTS, postural orthostatic tachycardia syndrome; APR, acute phase reactant; GPCR, G-protein-coupled receptors; AABs, autoantibodies; EBV, Epstein–Barr virus; IgG, immunoglobulin G; IgM, immunoglobulin M; IEA, intermediate early antigen; EA, early antigen.

## Data Availability

The data presented in this study are available on request from the corresponding author due to privacy or ethical restrictions.

## Supplementary Materials

The following supporting information can be downloaded at: https://www.mdpi.com/article/10.3390/medicina62030480/s1, Supplementary File: Appendix B: Assessment Protocol of the HALE study.

## Abbreviations

The following abbreviations are used in this manuscript:

AAB	Autoantibody
APR	Acute phase reactant
AT1R	Angiotensin II type 1 receptor
ß1-adr-R	ß-1 adrenergic receptor
ß2-adr-R	ß-2 adrenergic receptor
BG	Blood group
BP	Blood pressure
CCC	Canadian Consensus Criteria
CI95%	95% confidence interval
COVID-19	Coronavirus disease 2019
CXCR3	Chemokine receptor CXCR3
EA-D	Early antigen-diffuse
EA-R	Early antigen-restricted
EBNA1	Epstein–Barr nuclear antigen-1
EBV	Epstein–Barr virus
ECG	Electrocardiogram
ELIA	Employers’ liability insurance association
ETAR	Endothelin type A receptor
GPCR	G-protein-coupled receptor
HALE	Health Care Workers Affected by Long COVID and Exhaustion/Fatigue
HE	Hospital employee
IEA	Intermediate early antigen
IgG	Immunoglobulin G
IgM	Immunoglobulin M
IOM	Institute of Medicine
IQR	Interquartile range
M3R	Muscarinic acetylcholine receptor M3
M4R	Muscarinic acetylcholine receptor M4
ME/CFS	Myalgic encephalomyelitis/chronic fatigue syndrome
MoCA	Montreal Cognitive Assessment
OD	Occupational disease
OH	Orthostatic hypotension
PAR1	Protease-activated receptor 1
PCR	Polymerase chain reaction
PCS	Post-COVID-19 syndrome
PCS-ME/CFS	Hospital employee with PCS and diagnosis of ME/CFS
PCS-non-ME/CFS	Hospital employee with PCS without diagnosis of ME/CFS
PEM	Post-exertional malaise
POTS	Postural orthostatic tachycardia syndrome
SARS-CoV-2 STROBE	Severe acute respiratory syndrome coronavirus Strengthening the Reporting of Observational Studies in Epidemiology
SD	Standard deviation
SFN	Small fiber neuropathy
VCA	Virus capsid antigen
VWF	Von Willebrand factor
VWF-Ac-0	VWF activity for blood group 0
VWF-Ac-AB	VWF activity for blood groups A, B and AB
VWF-Ag-0	VWF antigen for blood group 0
VWF-Ag-AB	VWF antigen for blood groups A, B and AB

## Appendix A

**Table A1:** All responses given by all participants during Canadian Consensus Criteria (CCC) assessment.

	Total *		CCC Fulfilled *	
	*n*	%	*n*	%
Total number	19	100	7	100
**1. Exhaustion/fatigue and worsening of condition after exertion**				
Significant extent of a new onset, otherwise unexplained, of persistent physical or mental fatigue, which leads to a significant reduction in activity level	16	84.2	7	100
Exhaustion, increase in severe feeling of illness and/or pain after exertion, with a delayed recovery period (usually more than 24 h, may take days)	12	63.2	7	100
Symptoms can be worsened by any type of physical or mental exertion or stress	13	68.4	7	100
**2. Sleep disorders**				
Difficulty falling asleep	6	31.6	5	71.4
Trouble sleeping through the night	13	68.4	5	71.4
Altered day–night rhythm	7	36.8	4	57.1
Sleep does not lead to recovery	12	63.2	6	85.7
**3. Pain**				
Joint pain	7	36.8	4	57.1
Muscle pain	10	52.6	5	71.4
Headache	10	52.6	5	71.4
**4. Neurological/cognitive manifestation**				
Impairment of the ability to concentrate and short-term memory	15	78.9	7	100
Difficulties with information processing	9	47.4	3	42.9
Word-finding disorders	15	78.9	7	100
Reading disorders	5	26.3	4	57.1
Perceptual and sensory disturbances	4	21.1	3	42.9
Disorientation or confusion	3	15.8	2	28.6
Movement coordination disorders	3	15.8	3	42.9
Symptoms of overload (relapses and/or anxiety) occur due to: too much information, too many sensory impressions (e.g., light, noise), too much stress	14	73.7	5	71.4
**5. Autonomic manifestation**				
Rapid changes in position (especially from lying to standing) lead to dizziness and/or “blackout”	10	52.6	5	71.4
Heart palpitations occur during position changes (POTS)	9	47.4	4	57.1
Dizziness and lightheadedness	8	42.1	6	85.7
Extreme pallor	3	15.8	1	14.3
Intestinal disorders (diffuse pain, burning, flatulence)	7	36.8	4	57.1
Bladder disturbances	2	10.5	2	28.6
Heart palpitations	14	73.7	5	71.4
Shortness of breath during light exertion	16	84.2	6	85.7
**6. Neuroendocrine manifestation**				
Disturbed body temperature adjustment	4	21.1	2	28.6
Sweating, feverish feeling	7	36.8	4	57.1
Heat or cold not well tolerated	5	26.3	1	14.3
Cold extremities (cold hands or feet)	5	26.3	3	42.9
Weight gain or abnormal appetite	5	26.3	1	14.3
Weight loss	4	21.1	0	0.0
Stress is harder to handle, stress leads to increased exhaustion and emotional insecurity	17	89.5	1	100
**7. Immunological manifestations**				
Painful lymph nodes	2	10.5	1	14.3
Recurrent sore throat	4	21.1	2	28.6
New allergies/existing allergies have changed	2	10.5	1	14.3
Flu-like symptoms or general feeling of illness	8	42.1	5	71.4
Hypersensitivity, intolerance of foods, medications, chemicals	5	26.3	3	42.9

* Percentages were initially calculated based on the entire HALE-cohort and subsequently for the total number of participants fulfilling the CCC.

**Table A2:** Occurrence and duration (days) of new symptoms following the initial SARS-CoV-2 infection.

Symptom/Complication	*n*	%	Mean [Days]	Median [Days]	SD [Days]
Fatigue	19	100	655.74	627	139.06
Anosmia	12	63.2	317.58	320.5	283.18
Ageusia	12	63.2	288.25	158	273.69
Rhinitis	9	47.4	286.89	23	341.46
Headache	15	78.9	489.13	537	309.25
Concentration disorders	18	94.7	565.56	603.5	200.18
Memory disorders	16	84.2	616.19	608.5	125.52
Sore throat	13	68.4	421.23	565	297.08
Fever	9	47.4	5.78	6	3.23
Cough	12	63.2	408.33	547.5	303.90
Dyspnea	12	63.2	597.08	602	143.86
Breathlessness	16	84.2	615.75	614	93.61
Palpitations	18	94.7	637.44	626	130.84
Limb pain	12	63.2	511.00	551	235.14
Unspecific pain	10	52.6	460.60	603.5	277.94
Heaviness of limbs	13	68.4	532.62	565	214.03
Weakness in the limbs	16	84.2	534.81	557	216.61
Insomnia	19	100	611.16	606	157.72
Dysesthesia	11	57.9	639.55	628	113.28
Brain fog	10	52.6	605.10	645	287.11
Anxiety	16	84.2	551.81	603.5	198.36
Mood swings	15	78.9	614.80	631	250.31
Hair loss	12	63.2	592.83	583	196.89
Skin alterations	7	36.8	639.00	625	183.21
Nausea	4	21.1	492.50	573.5	212.47
Vomiting	1	5.3	582.00	582	
Diarrhea	6	31.6	534.50	556	289.07
Abdominal pain	9	47.4	598.89	614	191.01
Bloating	7	36.8	638.86	628	91.20
Diffuse bleeding	1	5.3	16.00	16	
Chest pain/tightness	11	57.9	607.55	628	152.09
Vertigo	13	68.4	619.69	582	184.07
Subfebrile temperature	4	21.1	701.25	703.5	147.71

New symptoms and complications following the initial SARS-CoV-2 infection reported in hospital employees. The duration of persisting symptoms is indicated by the duration from onset to end of appearance or the date of the assessment. Percentages are calculated for the entire HALE-cohort (*n* = 19). The duration of symptoms and complications is stated in days. SD, standard deviation.

**Table A3:** Full results of the coagulation panel and determination of autoantibodies (AAB) against G-protein-coupled receptors (GPCR).

	Unit	*n*	Median	Min	Max	% Low	% High	Reference Range
Coagulation								
Quick	%	18	105	81	124	0.0	0.0	70–130
INR	ratio	18	1.00	0.90	1.20	/	/	/
PTT	sec.	18	27.05	22.30	32.60	5.6	0.0	23–36
Thrombin time	sec.	18	14	12	15	0.0	0.0	10–17
Fibrinogen	mg/dL	18	402	325	548	0.0	16.7	276–471
Antithrombin	%	18	101.5	64	130	11.1	5.6	83–128
Factor VIII	%	18	134.5	75	174	0.0	16.7	50–150
Factor IX	%	18	105	66	156	0.0	5.6	65–150
Factor XIII antigen	%	18	114.5	82	163	0.0	5.6	75.2–154.8
Protein C	%	18	120.5	81	167	0.0	27.8	70–140
Protein S	%	18	106.5	55	150	5.6	11.1	63.5–149
APC resistance	ratio	18	2.90	2.80	3.10	0.0	0.0	2.4–3.5
VWF activity BG 0	%	18	119.5	59	215	0.0	38.9	40–126
VWF activity BG A, B, AB	%	18	119.5	59	215	0.0	16.7	49–163
VWF antigen BG 0	%	18	149	73	237	0.0	55.6	42–141
VWF antigen BG A, B, AB	%	18	149	73	237	0.0	22.2	66–176
Lupus anticoagulant (dRVVT)	ratio	18	1.00	0.90	1.10	/	0.0	0.0–1.2
Homocysteine	μmol/L	9	13.00	7.49	14.90	/	77.8	<10
GPCR AAB								
ß1-adr-R AAB	U/mL	18	7.20	2.60	59.70	/	16.7 *	<15.0
ß2-adr-R AAB	U/mL	18	11.75	2.50	73.10	/	66.7 *	<8.0; 8.0–14.0 ^†^
M3R AAB	U/mL	18	8.40	4.50	31.90	/	88.9 *	<6.0; 6.0–10.0 ^†^
M4R AAB	U/mL	18	7.65	4.40	33.00	/	22.2 *	<10.7
ETAR AAB	U/mL	18	4.61	1.45	40.00	/	5.6 *	<10.0; 10.0–16.9 ^†^
AT1R AAB	U/mL	18	5.86	3.69	40.00	/	22.2 *	<10.0; 10.0–16.9 ^†^
PAR1 AAB	U/mL	18	2.40	1.30	34.00	/	5.6 *	<13.0; 13.0–18.9 ^†^
CXCR3 AAB	U/mL	18	6.35	2.90	80.00	/	5.6 *	<30.0; 30.0–39.9 ^†^

A total of 19 participants were enrolled; one participant completed the assessment but declined blood sampling, resulting in a sample size of *n* = 18 for this analysis. Homocysteine levels were determined in nine participants only, as this parameter was not assessed in all participants.* Weak positive and positive values were considered as elevated above the reference range. ^†^ The first cut-off indicates the upper normal limit; the second range indicates weak positive values. INR, International Normalized Ratio; PTT, Partial Thromboplastin Time; APC resistance, activated protein C resistance; VWF, von Willebrand factor, dRVVT, normalized dilute Russell’s viper venom time ratio; BG, blood group; ß1-adr-R, ß-1 adrenergic receptor; ß2-adr-R, ß-2 adrenergic receptor; M3R, muscarinic acetylcholine receptor M3; M4R, muscarinic acetylcholine receptor M4; ETAR, endothelin type A receptor; AT1R, angiotensin II type 1 receptor; PAR1, protease-activated receptor 1; CXCR3, chemokine receptor CXCR3.

**Table A4:** Full Epstein–Barr virus serology.

		Positive				Negative	
		Weak Positive		Positive			
	Antigens	*n*	%	*n*	%	*n*	%
IgG	EBNA1	1	73.3	11	6.7	3	20.0
	p18 VCA	0	0.0	15	100.0	0	0.0
	p23 VCA	0	0.0	15	100.0	0	0.0
	BZLF1 * IEA	3	20.0	9	60.0	3	20.0
	p138 EA-R	1	6.7	1	6.7	13	86.7
	p54 EA-D	1	6.7	2	13.3	12	80.0
IgM	p23 VCA	0	0.0	0	0.0	15	100.0
	ZEBRA ^†^ IEA	0	0.0	0	0.0	15	100.0
	p138 EA-R	0	0.0	1	6.7	14	93.3
	p54 EA-D	1	6.7	0	0.0	14	93.3

Complete results of Epstein–Barr virus serology in the HALE-cohort. Serological testing was performed in participants who underwent the assessment at the Department of Occupational Medicine (*n* = 15). * Complete ZEBRA protein as antigen. ^†^ Immunodominant peptide fragment of the ZEBRA protein as antigen. IgG, immunoglobulin G; IgM, immunoglobulin M; EBNA1, Epstein–Barr nuclear antigen-1; VCA, virus capsid antigen; IEA, intermediate early antigen; EA-R, early antigen-restricted; EA-D, early antigen-diffuse.

**Table A5:** Full results of general blood work.

	Unit	N	Median	Min	Max	% Low	% High	Reference Range
Leukocytes	/nl	15	5.80	4.10	9.90	0.0	6.7	3.5–9.8
Erythrocytes	/pl	15	4.70	3.80	5.20	80.0	20.0	f 3.7–4.8 m 4.2–5.6
Hemoglobin	g/dL	15	14.10	10.90	15.10	6.7	13.3	f 11.3–14.5 m 13.3–16.2
Hematocrit	%	15	41.30	33.20	45.40	6.7	13.3	f 34–44 m 39–49
MCV	fl	15	88	81	95	13.3	0.0	83.2–99.8
MCH	pg	15	29.10	27.90	31.70	0.0	0.0	27.3–33.0
MCHC	g/dL	15	33.30	32.20	34.80	0.0	0.0	31.8–34.9
RDW	%	15	12.70	11.90	13.70	73.3	0.0	13.1–15.0
Thrombocytes	/nl	15	285	206	361	0.0	6.7	140–360
Basophils	%	15	0.80	0.30	1.60	/	6.7	<1.5
Eosinophils	%	15	2.00	0.90	4.80	0.0	0.0	0.4–6.6
Neutrophils	%	15	61.80	39.20	71.40	13.3	0.0	43.0–74.8
Lymphocytes	%	15	30.00	20.00	48.60	0.0	6.7	16.4–45.0
Monocytes	%	15	7.20	5.30	10.70	0.0	40.0	2.6–8.2
Urea	mg/dL	15	26.77	17.47	42.62	0.0	0.0	17–43
Creatinine	mg/dL	15	0.74	0.62	1.06	0.0	0.0	f 0.59–0.95 m 0.67–1.17
Glucose	mg/dL	12	81.5	58	105	8.3	8.3	60–100
Cholesterol	mg/dL	15	212	128	300	/	53.3	<200
Triglycerides	mg/dL	15	107	38	551	/	33.3	<150
HDL-cholesterol	mg/dL	15	57.70	43.80	74.30	60.0	/	>60
LDL-cholesterol	mg/dL	14	123	53	202	/	35.7	<150
AST	U/L	15	24	17	40	0.0	20.0	f < 35 m < 50
ALT	U/L	15	31	10	62	0.0	26.7	f < 35 m < 50
GGT	U/L	15	23	13	75	0.0	26.7	f < 38 m < 55
CK	U/L	15	104	62	167	0.0	0.0	f < 170 m < 190
TSH basal	µU/mL	15	1.28	0.90	3.90	0.0	0.0	0.35–4.5
fT3	pg/mL	15	2.96	2.55	3.16	0.0	0.0	2.3–4.2
fT4	ng/dL	15	0.99	0.82	1.16	20.0	0.0	0.89–1.76
Iron	µg/dL	15	98.00	33.90	193.50	6.7	6.7	f 39–149 m 35–168
Ferritin	ng/mL	15	111.20	10.30	484.40	0.0	6.7	f 10–291 m 22–322
Transferrin	g/L	15	2.82	2.37	3.76	0.0	0.0	f 1.80–3.82 m 1.74–3.64
Transferrin saturation	%	15	26	8	37	13.3	0.0	16–45
Vitamin D-25-OH	ng/mL	15	21.10	7.10	43.80	93.3	0.0	41–100

Full results of general blood work in the HALE-cohort. Blood analyses were performed in participants who completed the assessment at the Department of Occupational Medicine (*n* = 15). Sample sizes vary between parameters due to missing values (*n* per parameter indicated in the table). F, female; m, male; MCV, mean corpuscular volume; MCH, mean corpuscular hemoglobin; MCHC, mean corpuscular hemoglobin concentration; RDW, red blood cell distribution width; HDL, high-density lipoprotein; LDL, low-density lipoprotein; AST, aspartate transaminase; ALT, alanine transaminase; GGT, gamma-glutamyl transferase; CK, creatine kinase; TSH, thyroid-stimulating hormone; fT3, free triiodothyronine; fT4, free thyroxine.
