# Supplementary material for: Assessment and Incidence Determination of Myalgic Encephalomyelitis/Chronic Fatigue Syndrome Following a SARS-CoV-2 Infection in a Prospective Cohort of Hospital Employees
Source: Medicina (Kaunas). 2026 Mar 3;62(3):480. doi: 10.3390/medicina62030480 (PMC13027803; doi:10.3390/medicina62030480)
Supplement: Supplementary file 1 [file medicina-62-00480-s001.zip › medicina-4125804-supplementary.pdf]

Appendix B: Assessment Protocol of the HALE study

The following protocol describes the workflow and principal steps of the HALE study. Its primary purpose is to systematically collect data on participants, characterize their symptoms, and implement a standardized procedure to exclude potential alternative causes of post-COVID syndrome (PCS) and possible myalgic encephalomyelitis/chronic fatigue syndrome (ME/CFS). The protocol provides an overview of the study procedures, while detailed descriptions are presented in the main manuscript.

1. **Telephone Interview**
    a. Informed consent
    b. General medical history since infection
    c. Presence of fatigue?
    d. Any additional new symptoms since infection?
2. **Assessment at the Institute of Hygiene**
    a. Written informed consent
    b. Questionnaire:
        i. General medical history since infection
        ii. Reinfection(s) with SARS-CoV-2?
        iii. COVID-19 vaccinations?
        iv. Symptoms (n = 33) with new onset after primary SARS-CoV-2 infection → presence and duration?
    c. Canadian Consensus Criteria (CCC) for the diagnosis of ME/CFS→ fulfilled?
    d. Bell-Score → functional status?
    e. ECG → abnormalities?
    f. Orthostatic Test → POTS? Orthostatic hypotension (OH)? Symptoms?
    g. Montreal Cognitive Assessment (MoCA test) → mild cognitive impairment?
    h. SARS-CoV-2 PCR testing by hypopharyngeal swab → positive?
    i. Screening for possible small fiber neuropathy (SFN)
    j. Blood sampling:
        i. Coagulation panel
        ii. GPCR AABs (blood samples initially frozen)
        iii. Thrombelastography
3. **Further Assessment** at the Department of Occupational Medicine/Primary Care Physician
    a. For further medical evaluation and the systematic exclusion of potential differential diagnoses of PCS, and where applicable ME/CFS, HEs directly employed by the hospital underwent additional assessment at the **Department of Occupational Medicine** according to this standardized protocol. HEs no longer employed by the hospital or working for

subcontractors were instructed to follow the same protocol through their **primary care physician**:

    i. Full medical history
    ii. Symptoms following SARS-CoV-2 infection?
    iii. Pre-existing conditions?
    iv. Current medications?
    v. Physical examination
    vi. Height, weight, vital signs?
    vii. Primarily affected organ systems?

b. **Blood samples** (Blood samples were required from all participants, wherepossible. Those collected by primary care physicians were used solely for exclusion purposes and not for standardized data collection, owing to differing laboratory standards):
    i. Complete blood count
    ii. Basic renal tests: Urea, creatinine, creatine kinase
    iii. Hepatic tests: AST, ALT, GGT
    iv. Metabolic function tests: glucose, cholesterol, triglycerides, HDL-cholesterol, LDL-cholesterol
    v. Thyroid hormones: basal TSH, fT3, fT4
    vi. Iron metabolism tests: iron, ferritin, transferrin, transferrin saturation
    vii. Vitamin D-25-OH
    viii. EBV antibody serology → Reactivation?
c. Only for employees participating in the occupational medicine evaluation:
    i. Should the participant’s condition be classified as an occupational disease (OD)?
    ii. Was the OD confirmed by the Employers' Liability Insurance Association (ELIA)?
    iii. Was treatment initiated by the ELIA?
    iv. Did the participant receive rehabilitation treatment?

4. **Re-evaluation** of previous results and determination of the need for further investigations in other **specialty departments**:
    a. Cardiological symptoms (e.g. palpitations, chest pain/tightness, ECG results, orthostatic test results) → Department of Cardiology
    b. Respiratory symptoms (e.g. dyspnea) → Department of Pneumology
    c. Neurological symptoms (e.g. concentration disorders, dysesthesia, positive SFN screening questions or poor results in the MoCA test) → Department of Neurology
    d. E.g. vertigo, anosmia, ageusia → Department of ENT
    e. E.g. abdominal pain, diarrhea → Department of Gastroenterology
    f. E.g. impaired vision → Department of Ophthalmology
    g. E.g. anxiety → Department of Psychosomatic Medicine

5. Interdisciplinary case conference
    a. All previous findings were collected and reviewed. Subsequently, a final evaluation was conducted to determine whether alternative explanations for the newly emerged symptoms existed and to establish whether the symptoms were consistent with PCS, and, where applicable, with ME/CFS.

Abbreviations: AAB, Autoantibody; CCC, Canadian Consensus Criteria; COVID-19, Coronavirus disease 2019; EBV, Epstein-Barr virus; ECG, Electrocardiogram; ELIA, Employer's liability insurance association; GPCR, G-protein coupled receptor; HALE, Health Care Workers Affected by Long COVID and Exhaustion/Fatigue; ME/CFS, Myalgic encephalomyelitis/chronic fatigue syndrome; MoCA, Montreal Cognitive Assessment; OD, Occupational disease; OH, Orthostatic hypotension; PCR, Polymerase chain reaction; PCS, Post-COVID syndrome; POTS, Postural orthostatic tachycardia syndrome, SARS-CoV-2, Severe scute respiratory syndrome coronavirus; SFN, Small fiber neuropathy.
